# Establishment of a seafood index to assess the seafood consumption in pregnant women

**DOI:** 10.3402/fnr.v57i0.19272

**Published:** 2013-02-28

**Authors:** Maria W. Markhus, Ingvild E. Graff, Lisbeth Dahl, Camilla F. Seldal, Siv Skotheim, Hanne C. Braarud, Kjell M. Stormark, Marian K. Malde

**Affiliations:** 1 NIFES (National Institute of Nutrition and Seafood Research), Bergen, Norway; 2 The Department of Biomedicine, Faculty of Medicine and Dentistry, University of Bergen, Bergen, Norway; 3 The Department of Medicine, Faculty of Medicine and Dentistry, University of Bergen, Bergen, Norway; 4 Regional Centre for Child and Youth Mental Health and Child Welfare, Uni Health, Uni Research, Bergen, Norway

**Keywords:** seafood consumption, marine omega-3 fatty acids, 25OH vitamin D, FFQ, biomarkers, pregnancy

## Abstract

**Background:**

Seafood (fish and shellfish) is an excellent source of several essential nutrients for pregnant and lactating women. A short food frequency questionnaire (FFQ) that can be used to quantitatively estimate seafood consumption would be a valuable tool to assess seafood consumption in this group. Currently there is no such validated FFQ in Norway.

**Objective:**

The objective of this study was to establish and validate a seafood index from a seafood FFQ against blood biomarkers (the omega-3 index, the omega-3 HUFA score, and serum 25OH vitamin D).

**Design:**

We assessed maternal seafood consumption during the 28th gestation week in healthy Norwegian women (*n*=54) with a seafood FFQ. A seafood index was developed to convert ordinal frequency data from the FFQ into numerical scale data. The following blood biomarkers were used as a validation method: omega-3 index, omega-3 HUFA score, and the serum 25OH vitamin D.

**Results:**

The reported frequency of seafood as dinner and as spread was strongly correlated with the estimated frequencies of seafood as dinner and as spread. This indicated that the seafood index is a valuable tool to aggregate reported frequencies from the seafood FFQ. The seafood index composed of the frequency of seafood consumption and intake of omega-3 supplements, termed the *total* seafood index, correlated positively with the omega-3 index, omega-3 HUFA score, and 25OH vitamin D.

**Conclusion:**

We established and validated a seafood index from a seafood FFQ. The developed seafood index can be used when studying health effects of seafood consumption in large populations. This seafood FFQ captures seafood consumption and omega-3 supplement intake considerably well in a group of pregnant women.

In nutritional epidemiology, the aim is usually to elucidate the relationships between diet and health. Recent evidence suggests that the intake of marine omega-3 polyunsaturated fatty acids (PUFAs) in pregnancy and early life play an important role in the growth and development of the foetus brain (1, 2). Seafood is a unique source of the marine omega-3 PUFAs eicosapentaenoic acid (EPA) and docosahexaenoic acid (DHA), in addition to vitamin D, vitamin B_12_, iodine, and selenium (3). However, seafood may also contain persistent organic pollutants and heavy metals in concentrations that are somewhat higher than food items like milk, meat, and eggs. The most sensitive life stage for exposure to undesirable substances is during foetal development, and, thus, the seafood intake in pregnant women is of particular interest. However, the general recommendation in Norway is to eat seafood 2–3 times a week, and with documented health effects, taken the generally low seafood consumption among young women into account, they would profit rather than lose in increasing their seafood consumption. Furthermore, based on knowledge about young women's consumption of fatty fish, there is no reason to believe that a general recommendation to increase fish consumption would result in fertile women consuming fatty fish to the extent that the intake of contaminants over a long period, would exceed the tolerable weekly intake and consequently constitute a potential health risk for the foetus (3). However, in any case pregnant women should follow the national guidelines and avoid the limited number of fish species containing high levels of methyl mercury (3).

In Norway, the recommended seafood consumption in a healthy balanced diet is 300–450 g of fish per week, of which at least 200 g should be from oily fish (>5% fat) (4). Other sources of omega-3 PUFAs such as flaxseed oil and rapeseed oil contain α-linoleic acid (ALA) that needs to be converted to longer-chain EPA and further to DHA to become biologically useful. Consuming omega-3 PUFAs as EPA and DHA is therefore preferable to other sources because the ability of the human body to synthesise EPA and DHA from ALA is limited. A certain conversion of ALA to EPA occurs; however, the conversion to DHA is severely restricted. ALA competes with omega-6 PUFAs and uses the same enzymes to convert to EPA and DHA. Western diets tend to be inadequate in omega-3 PUFAs and rich in omega-6 PUFAs leading to a decreased synthesis of EPA and DHA (5). Furthermore, there is evidence that fatty acid metabolism could be impaired in some individuals (6).

Prospective cohort studies, assessing the habitual diet, offer the best opportunity to gather valid and reliable information about nutritional status (7). A short food frequency questionnaire (FFQ) may be adequate to assess seafood consumption and investigate the associations between seafood consumption and health. In contrast to a standard FFQ measuring the whole diet, a short FFQ is less demanding for the participants to complete, can be more detailed, and repeated more frequently. Short FFQs are also cheaper to administrate than other traditional dietary assessment methods. The time used for data processing is reduced compared to other methods (8). Maternal food consumption can change considerably within a short period. Consequently, the advantages of a short FFQ are highlighted when assessing the effect of seafood consumption on health during pregnancy and lactation, when repeated monitoring of the diet is advisable.

Biomarkers can be objective measures of certain aspects of a dietary intake and can thus be used to validate the performance of a dietary assessment method (7). Biomarkers can also be used separately as alternatives for conventional dietary assessment methods. Omega-3 PUFA levels in blood have previously been shown to predict dietary habitual fish consumption (9, 10). PUFA status in red blood cell (RBC) membranes reflects the recent 3 months; offering a more aggregated time period than serum or plasma. Thus, RBC is regarded a more suitable tissue than adipose tissue to validate a FFQ in pregnancy, as the PUFA status in RBC reflects the recent months and not the previous year (11, 12). The omega-3 index is the content of EPA and DHA in the RBC membranes, expressed as a percentage of total fatty acids (FAs) and optimal levels appear to be 8% or greater (13). In addition to the omega-3 index, the proportion of highly unsaturated omega-3 fatty acids (HUFAs) in tissues can also be used as an indicator of disease risk (14). The omega-3 index was originally suggested as a marker of increased risk of death from coronary heart disease, but it can also be viewed as an actual risk factor, playing a pathophysiologic role in the disease (13). This study concurs with that of Harris, that using the omega-3 index in the design of studies might allow for a more efficient use of research resources as results from studies will be easier to compare and contrast (15). 25OH vitamin D_3_ is the lipophilic vitamin D's main transported metabolite with a half-life of approximately 15 days (16). Serum 25OH vitamin D_3_ is the biomarker for vitamin D status with contributions both from dietary vitamin D and vitamin D produced from sun exposure (17). In high latitudes (i.e. Scandinavia), there are seasonal differences in vitamin D status due to a relatively low vitamin D intake in general, and limited sun exposure during the winter.

The nutrient intake of an individual is often calculated using the reported consumption frequency per day, multiplied by portion size in grams (or standard portion size), multiplied by nutrient content per 100 g and divided by 100 (18). To date, the Norwegian food composition table only reports the sums of saturated, monounsaturated, or polyunsaturated fats, and not individual FAs in foods (19). A different approach is therefore needed when studying the associations between the intake of marine omega-3 PUFAs and health. A validated seafood index could also be valuable as a screening tool to assess seafood consumption in antenatal care.

The objective of this study was to establish and validate a seafood index from a seafood FFQ against blood biomarkers (the omega-3 index, the omega-3 HUFA score, and serum 25OH vitamin D).

## Materials and methods

### Study population and design

The study population for this cross-sectional validation study was from a prospective cohort studying the associations between seafood consumption, mental health, and infant development. The target population included all pregnant women who gave birth within the period 2010–2011, in a municipality outside Bergen, Norway. Midwives and medical doctors recruited pregnant women from September 2009 until June 2011 at a routine visit during the 24th gestation week. There were no exclusion criteria for participation. At the routine visit in the 28th gestation week, non-fasting venous blood was drawn from the participants. The seafood FFQ was administered electronically using ‘Questback.com’. A link to the questionnaire and a unique identification number was e-mailed to all participants around the 28th gestation week and the participants completed it online around the 28th–32nd gestation week. The researchers subsequently downloaded data from Questback. Written informed consent was obtained from all volunteers at their routine visit during the 24th or 28th gestation week. Participants could withdraw from the study at any time, without reason. The procedures followed were in accordance with the Helsinki Declaration of 1975, as revised in 2008, and approved by the Regional Committee of Ethics in Medical Research West and the Norwegian Social Science Data Services. A biobank was established and approved for storage of biological samples.

### Dietary assessment

**Fig. 1:**
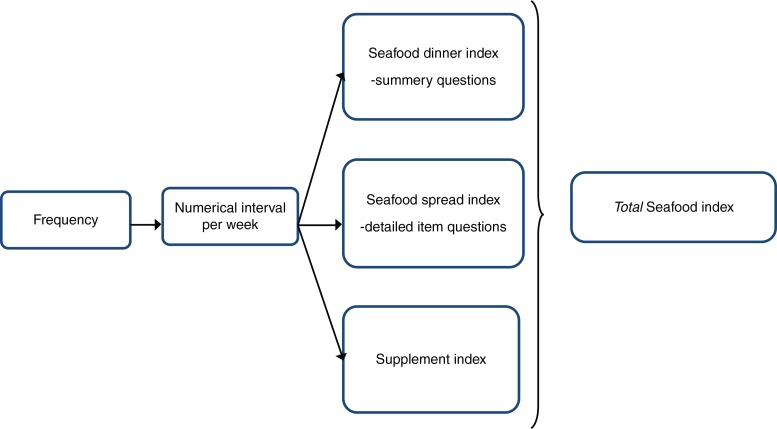
Flow chart of the seafood index.

The seafood FFQ is a semi-quantitative short questionnaire designed to capture the habitual intake of seafood (fish and shellfish) and the use of dietary supplements (20). In the first section, the respondents were asked to fill in the average frequency of food items eaten since the first gestation week. Questions regarding seafood intake included two summary questions concerning the frequency of eating seafood as dinner and as spread. Seafood as spread consisted of seafood as sandwich spread, in salads, and as snack meal. There were 24 detailed item questions regarding dinner and 13 questions regarding spread, focusing on type of seafood species and products (Fig. 1). Open spaces for items not included in the list allowed for a better characterisation of the participant's diet. The frequency intervals ranged from never to more than three times a week for dinner related questions, and from never to more than five times a week for questions regarding spread. The maximum frequency for spread was higher than for dinner due to a more frequent intake of bread in Norway. Regarding portion sizes, the respondent could choose between one out of five sizes ranging from half a portion to three portions. One portion was specified as a standard Norwegian dinner size of 150 g (21). The portion size was used to calculate weekly seafood consumption for dinner (g/week).

Data on the use of supplements (fish oil/omega-3 supplements, multivitamins and minerals, iron, B-vitamins, calcium, vitamin D, and ‘other’) had frequencies from never to daily. For participants using liquid or encapsulated fish oil, the questionnaire differentiated between taking this supplement over the whole year or just during the winter. The amount of fish oil taken was defined as a teaspoon (3 ml), a child's spoon (5 ml) or a tablespoon (10 ml), whilst the amount of capsules taken were defined as one to two, three to four, or five or more. The second section of the seafood FFQ covered questions regarding other important dietary sources of PUFAs and vitamin D, such as butter and margarine, and the use of fats for cooking. Subsequent questions focused on important non-dietary vitamin D sources and factors affecting vitamin D status, that is, ethnicity and sun exposure. Dairy products, together with seafood, are the most important iodine sources in the Norwegian diet and, thus, frequency questions regarding dairy products were included. In addition, we included questions regarding the consumption frequency of other food items (such as fruit and vegetables), lifestyle, and anthropometric and demographic factors. The body mass index (BMI) was calculated from reported height and weight.

### The seafood index

Figure 1 shows a flow chart of the seafood index. A numerical interval and a following seafood index were defined based on the respondents reported average frequency of seafood as dinner and seafood as spread (Table 1). Ordinal data from the seafood FFQ were converted into numerical data, according to the seafood index. In this way, aggregation of different types of seafood and quantity estimation of seafood consumption from a short FFQ were made possible. When asked about consumption frequencies of several detailed species or products separately, recall is prone to over-reporting on low intakes (22, 23). The seafood index was therefore established based on the lowest possible weekly intake of seafood items eaten as dinner and as spread (Table 1). The seafood indexes for dinners were grouped into five categories comprising dinner items of oily fish, lean fish, shellfish, processed fish, and freshwater fish. Freshwater fish consumption was divided into two separate questions, frequency of perch/pike (lean fish) and frequency of char/whitefish (oily fish). These two separate questions were also grouped into either lean or oily fish, respectively. This is due to the possible mercury pollution of Norwegian freshwaters and certain freshwater fish (24). The seafood FFQ therefore enabled the study of freshwater fish consumption. The reported intake of seafood as spread was grouped in oily or lean spread. In Norway, shrimps are normally eaten both for dinner (as spread) and as spread. The respondent could report average frequency of shrimps for dinner. However, there was no item question regarding the average frequency intake of shrimps as spread. The item question ‘other spread’ was mainly specified by the respondents as shrimp. To avoid double reporting on shrimp consumption, the seafood index for shrimps for dinner and the seafood index for ‘other spread’ were halved. Processed fish foods typically consist of 40–60% fish fillet (3). The lowest possible weekly intake of processed seafood was therefore halved prior to calculating the index for processed seafood as dinner. Missing answers were regarded as ‘never consumed’ when the seafood index was calculated.

**Table 1:** The seafood index – conversion of frequencies to numerical values

Reported frequency	Numerical interval per week†	Seafood index (summary question)‡	Seafood index (detailed item question)§
Never	0	0	0
<1 time/month	>0–0.25	0.15	0.1‖
1–3 times/month	0.25–0.75	0.50	0.25
1–2 times/week	1–2	1.5	1
≥3 times/week	≥3	3	3

† Numerical interval based on the average weekly frequency of seafood intake for dinner and seafood intake as spread.

‡ Seafood index assigned the average weekly frequency of seafood intake for dinner and seafood intake as spread.

§ Seafood index based on the lowest possible weekly intake of items eaten as dinner and items eaten as spread.

‖ Numerical value set to 0.1 to enable distinction between the two lowest frequencies.

Omega-3 supplement intake was re-coded from general frequency to frequency of average weekly intake to correlate and estimate intake in conjunction with the seafood intake. A numerical index was assigned, similar to that for seafood (Table 2).

**Table 2:** The supplement index – conversion of frequencies to numerical values

Reported frequency	Numerical interval per week†	Supplement index‡
Never	0	0
1–3 times/month	0.25–0.75	0.5
1–3 times/week	1–3	2
4–6 times/week	4–6	5
Daily	7	7§

† Numerical interval based on the average weekly intake of supplements.

‡ Supplement index based on the mean weekly intake of supplement.

§ When calculating the total seafood index, the highest frequency is set to the same as for the second highest frequency to correspond to the frequency range in the two main questions (seafood as dinner and seafood as spread).

An index termed the *total* seafood index was computed as a sum of the participants’ seafood dinner index, seafood spread index, and supplement index (Fig. 1). For example, a seafood dinner index of 1, seafood spread index of 1, and a supplement index of 5 resulted in a *total* seafood index of 7. The range of the *total* seafood index was 0–11.

### Verification of the seafood index

The sum of the seafood indexes for all detailed dinner items and the sum of the seafood indexes for all detailed spread items was termed the *estimated* dinner index and the *estimated* spread index, respectively. Theoretically, the corresponding frequency to the *estimated* dinner index should equal the corresponding frequency to the seafood dinner index, and the corresponding frequency to the *estimated* spread index should equal the corresponding frequency to the seafood spread index (Table 3). For example, a reported intake in the summary question of seafood as dinner 1–2 times per week, specified in the detailed item question as salmon 1–3 times per month, cod 1–3 times per month, fishcakes 1–2 times per week, and fish balls 1–2 times per week resulted in an *estimated* seafood dinner index of 1.5 and a seafood dinner index of 1.5.

$$\text{0.25}+\text{0.25}+\left(\frac{\text{1}+\text{1}}{\text{2}}\right)=1.5$$

**Table 3:** Frequency estimates from the seafood index

Interval†	Frequency estimate
0–0.14	Never
0.15–0.45	<1 time/month
0.5–0.95	1–3 times/month
1.0–2.9	1–2 times/week
3‡→	≥3 times/week

† Sum of type of seafood or seafood product.

‡ Maximum sum is set to 3.

### Laboratory analyses

Venous blood from the antecubital vein was collected in ice water cooled 4 ml BD Vacutainer^®^ K2E 7.2 mg vials and centrifuged (1,000–1,300 G, 20°C, 10 min) within 30 min for the preparation of RBC. Venous blood for serum preparation was collected in 3.5 ml BD Vacutainer^®^ SST™ vials II *Advanced*, set to coagulate for minimum 30 min and centrifuged (1,000–1,300 G, 20°C, 10 min) within 60 min. RBC and serum samples were stored at −20°C for 0–4 weeks prior to transportation on dry ice to −80°C storage until analysis. RBCs were adequately separated to ensure a clean blood fraction.

FA composition of total RBC was determined by ultrafast gas chromatographic (UFGC) (Thermo Electron Corporation, Massachusetts, USA), a method developed by Araujo et al. (25). After direct methylation of FAs in homogenised samples, boron trihalide and internal standard (19:0 methyl ester) were added, followed by extraction with hexane. The FA composition was calculated using an integrator (Chromeleon 6.80, Dionex Corporation, California, USA) connected to the UFGC and identification ascertained by standard mixtures of methyl esters (Nu-Chek, Minnesota, USA). Limit of quantification was 0.01 mg FA/g samples (wet weight). The analytical quality of the method and systematic errors were controlled by the certified reference materials (CRM) 162 (soy oil) and 163 (pig fat).

The omega-3 HUFA score is the percentage of omega-3 HUFAs in the total RBC (or other tissue) HUFA pool and has been described previously as a biomarker of omega-3 FAs in tissues (26, 27).

The serum 25OH vitamin D concentration was determined by a modified version of Kissmeyer and Sonne using a liquid chromatographic-tandem mass spectrometric (LC-MS/MS) assay adding acetonitril and internal standard (^2^H 25OH vitamin D_3_) to the samples (28). Samples were cleansed using solid phase extraction columns and solvents were evaporated with nitrogen after elution. The samples were then dissolved in a buffer mixture, and quantified using Quanlynx. The limit of quantification was 0.25 ng/ml. The analytical quality of the method and systematic errors were controlled by the CRM 2972 (calibration solution) and CRM 972 (human serum). Only those participants who answered in the winter (October–April) were assessed for the association between seafood intake and the serum 25OH vitamin D concentration, due to the endogenous synthesis of 25OH vitamin D from sunlight during the summer (29, 30).

### Statistical methods

Means and standard deviations (SD) were calculated for the normally distributed biomarkers, and median (range) were calculated for the biomarkers that were not normally distributed. Normality of the biomarkers was tested with Kolomogorov–Smirnov statistics. An independent sample *t*-test for normally distributed data and Mann–Whitney U-test for non-normally distributed data were used to examine the difference between biomarker status in supplement users and non-supplement users. The Spearman's rank correlation was used to measure the strength of association between two variables of non-parametric variables. Comparisons between several groups were tested with one-way analysis of variance (ANOVA) for normally distributed data and the Kruskal–Wallis test for non-parametric data. Correlation coefficients between dietary methods are considered good, if above 0.50, fair, if 0.30–0.49, and poor, if <0.30 (31). All statistical analyses were carried out using the Statistical Package for the Social Sciences (IMB^®^ SPSS^®^ Statistics 19, IBM Corporation, Norway).

## Results

A total of 55 (76%) participants answered the seafood FFQ and gave blood samples. Their average age was 29.6±4.8 years, and their average BMI was 23.6±3.9. The education level was high, and the income was average for Norway (Table 4).

**Table 4:** Socio-economic and behavioural characteristics of study participants (*n*=55)

Characteristics	Count	Percent
Education		
Lower secondary school	0	0
Higher secondary school	16	29.1
< 4 years of university education†	24	43.6
≥4 years of university education†	15	27.3
Employment		
Full-time (80–100%)	48	87.3
Part-time (50–79%)	2	3.6
Part-time (<50%)	1	1.8
Homemaker	1	1.8
Other	3	5.5
Marital status‡		
Married	23	43.4
Cohabiting	28	52.8
Single	2	3.8
Own income in NOK§		
< 1,50,000	2	3.6
1,50,000–1,99,999	1	1.8
2,00,000–2,99,999	12	21.8
3,00,000–3,99,999	27	49.1
4,00,000–4,99,999	10	18.2
> 5,00,000 NOK	3	5.5
Self-reported smoking during pregnancy‡		
Non-smoker	52	98.1
Current smoker	1	1.9
Self-reported use of snuff during pregnancy‖		
Non-user	54	100
Current user	0	0

† University or University College.

‡ *n*=53.

§ 1,00,000 NOK 14,000 EUR.

‖ *n*=54.

In this study, approximately 71% of the participants ate seafood for dinner, 1–2 times per week, and the prevalence of consuming seafood as spread 1–2 times per week was 36%. The estimated median intake of seafood as dinner was 32 (3–64) g per day. Daily omega-3 supplementation was reported by 56% of the participants, and 7% reported using omega-3 supplement more than 4 days per week. All the reported omega-3 supplements contained vitamin D.

Summary statistics for biomarkers are presented in Table 5, for all participants and non-supplement users; the latter group was separated from the whole study population to be able to assess the effect of seafood intake as a food matrix alone.

**Table 5:** Summary statistics for biomarkers in all participants (*n*=55) and non-supplement users (*n*=19)

	All participants		Non-supplement users only	
		
Biomarker	Mean±SD	Min, max	Mean±SD	Min, max
25OH vitamin D* (nmol/L)†	71±30	23, 148	54±22¶	23, 98
EPA (mg/g RBC)‡	0.025±0.02	0.0, 0.07	0.017±0.01¶	0.0, 0.06
DPA (mg/g RBC)§	0.052±0.01	0.02, 0.08	0.050±0.01	0.02, 0.08
DHA (mg/g RBC)‡	0.17±0.04	0.0, 0.25	0.16±0.04	0.09, 0.25
Omega-3 index§	6.9±1.7	3.5, 11.0	6.4±1.6	3.7, 10
RBC HUFA score‖	37±6	23, 52	34±5¶	28, 49

* Vitamin D refers to serum 25OHD3 concentration.

† All participants: *n*=51, non-supplement users only: *n*=18.

‡ All participants: *n*=54.

§ The content of EPA+DHA in red blood cells membranes expressed as a percent of total fatty acid (13).

‖ Total HUFA is the sum of the omega-3 and the omega-6 HUFAs, and the red blood cells omega-3 HUFA score equals 100% – omega-6 HUFA (14).

¶ Differences between supplement users and non-supplement users are statistically significant at the level 0.05. Results from supplement users are not shown.

Non-supplement users have a lower serum 25OH vitamin D concentration, a lower EPA concentration in the RBC, and a lower RBC omega-3 HUFA score than supplement users. However, the concentration of DHA in the RBCs and the omega-3 index did not differ between these two groups (Table 5).

### Verification of the seafood index

**Fig. 2:**
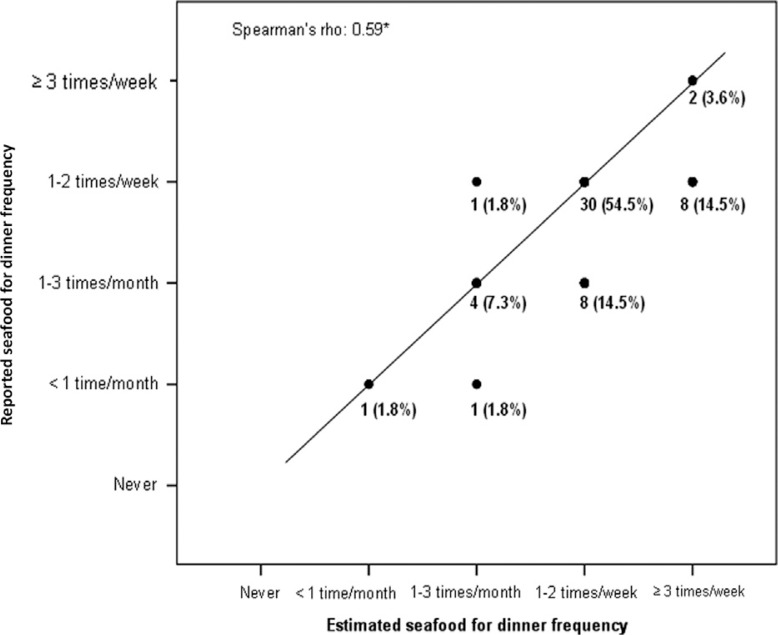
The figure illustrates the association between the reported seafood for dinner frequency (summary question) and the *estimated* seafood for dinner frequency (based on seafood index from all dinner items). The sum of points equals 100. The line represents matching frequencies. Spearman's rho between the reported seafood dinner frequency and the *estimated* seafood dinner frequency was 0.59, with a significance level of *P*>0.001.

**Fig. 3:**
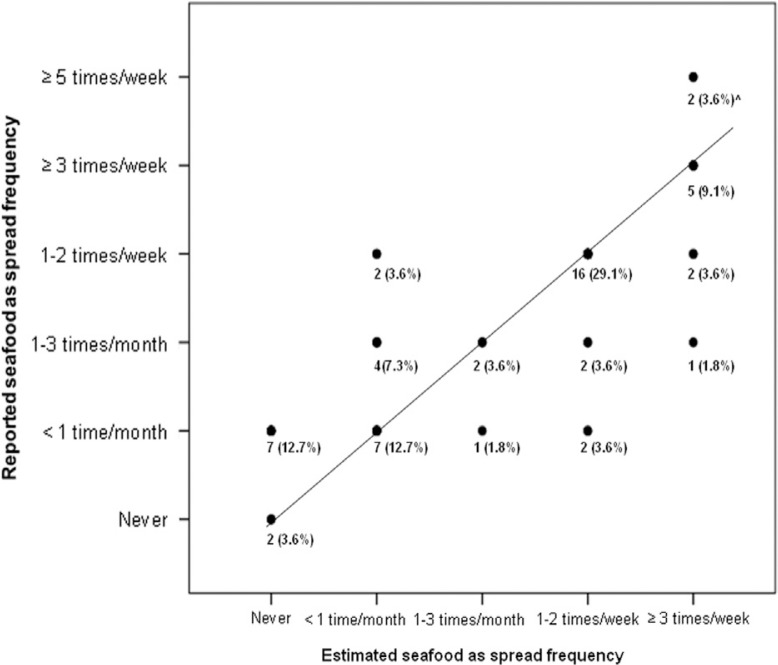
The figure illustrates the association between the reported seafood as spread frequency (summary question) and the *estimated* seafood as spread frequency (sum of all seafood as spread items). The sum of points equals 100. The line represents matching frequencies. Spearman's rho between the reported seafood as spread frequency and the *estimated* seafood as spread frequency was 0.82, with a significance level of *P*>0.001. ∧The data points could be allocated with the data points below as the *estimated* seafood as spread frequency was maximum ≥3 times/week.

Figures 2 and 3 illustrate the strong positive correlation between the reported seafood for dinner and as spread frequency, and the *estimated* seafood for dinner and as spread frequency.

There were strong positive correlations between the seafood dinner index and the *estimated* seafood dinner index, and between the seafood spread index and the *estimated* seafood spread index (Spearman's rho =0.60, *P*<0.001, and Spearman's rho =0.81, *P*<0.001, respectively).

### Correlations between seafood indexes and biomarkers

Correlations between the seafood indexes and the biomarkers are presented in Table 6. There was a positive correlation between the oily fish dinner index and the serum 25OH vitamin D concentration (Spearman's rho=0.37, *P*<0.05) (Table 6). We also found a positive correlation between the supplement index and the serum 25OH vitamin D concentration (Spearman's rho=0.62, *P*<0.005) (Table 6). A positive correlation was also found between the spread index and the three biomarkers: DHA concentration in RBCs, the omega-3 RBC HUFA score, and the omega-3 index. There were no statistically significant correlation between the seafood dinner index, the lean fish index, or the processed fish index, and the biomarkers (results for the latter are not shown).

**Table 6:** Spearman's rho coefficients of the association between the different seafood indexes and biomarkers in all participants, *n*=54

	Seafood dinner indexes†		Seafood spread indexes‡				
				
Biomarker	Oily fish dinner index	Lean fish dinner index	Spread index	Oily fish spread index	Lean fish spread index	Supplement index§	Total Seafood index¶
25OHD_3_ (nmol/L)‖	0.37*	0.33	−0.09	0.27	−0.05	0.62**	0.44*
EPA (mg/g RBC)	0.09	0.05	0.27	0.28*	0.12	0.37**	0.53**
DPA (mg/g RBC)	0.14	0.0	0.22	0.21	0.14	0.19	0.34*
DHA (mg/g RBC)	0.07	−0.08	0.33*	0.39**	0.03	0.22	0.37**
Omega-3 index#	0.15	0.01	0.28*	0.32*	0.17	0.21	0.36**
RBC omega-3 HUFA score††	0.13	0.03	0.35**	0.41**	0.11	0.35**	0.49**

† The seafood dinner indexes were defined based on the respondents reported average frequency of seafood eaten as dinner. Seafood as dinner was grouped into five categories oily, lean (results in table), processed, shellfish and freshwater fish (results not in table).

‡ The seafood spread indexes were defined based on the respondents reported average frequency of seafood eaten as spread. Seafood as spread was grouped into two categories (oily and lean).

§ The supplement index was defined based on the respondents reported average frequency of omega-3 supplement intake.

¶ The *total* seafood index was computed as a sum of the participants’ seafood dinner index, seafood spread index and supplement index.

‖ Participants who answered in the winter only *n*=30.

# The content of EPA+DHA in red blood cells membranes expressed as a percent of total fatty acid (13).

†† Total HUFA is the sum of the omega-3 and the omega-6 HUFAs, and the red blood cells omega-3 HUFA score equals 100% – omega-6 HUFA (14).

*/** Indicates statistical significance at the 0.05 or 0.001 level, respectively.

**Fig. 4:**
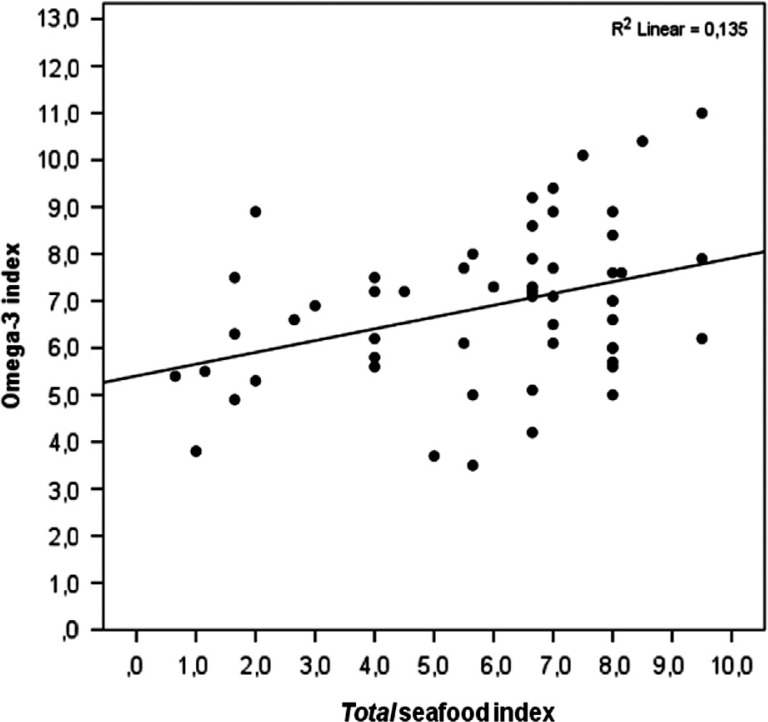
The association between the *total* seafood index (fish, other seafood, and omega-3 supplement intake) and the omega-3 index, in all participants (*n*=54).

**Fig. 5:**
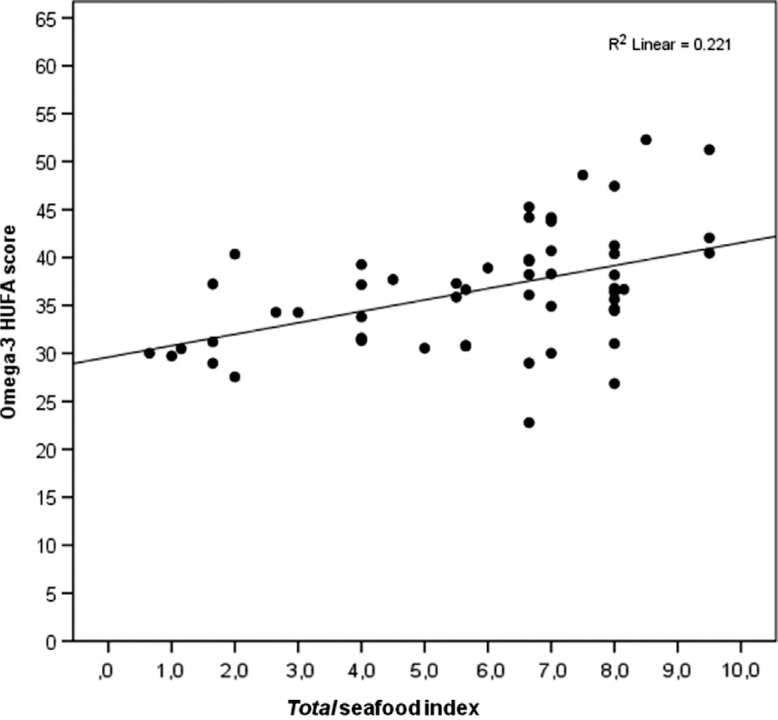
The association between the *total* seafood index (fish, other seafood, and omega-3 supplement intake) and the omega-3 HUFA score, in all participants (*n*=54).

**Fig. 6:**
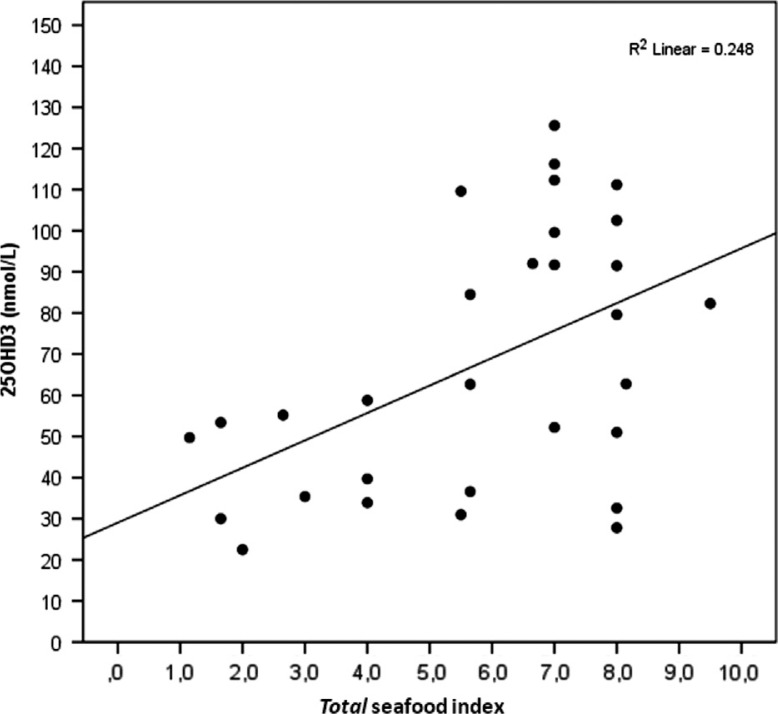
The association between the *total* seafood index (fish, other seafood, and omega-3 supplement intake) and serum 25OH vitamin D, in participants who answered in the winter months (October–April) (*n*=30).

The *total* seafood index correlated positively with the omega-3 index in all participants (R^2^=0.135, Fig. 4), with a rank correlation of 0.36 (*P*<0.005, Table 6). The correlation between the *total* seafood index and the RBC omega-3 HUFA score (R^2^=0.22, Fig. 5) was even stronger than between the *total* seafood index and the omega-3 index, with a rank correlation of 0.49 (*P*<0.005, Table 6). A positive correlation was found between the *total* seafood index and the serum 25OH vitamin D concentration of the participants who answered in the winter (R^2^=0.248, Fig. 6), with a rank correlation of 0.44 (*P*<0.05, Table 6).

Biomarker concentrations increased across increasing quartiles of the *total* seafood index (Table 7). Participants with a *total* seafood index in the upper quartile (>8–11) had a significantly higher biomarker status, including all the biomarkers analysed, than participants with a *total* seafood index in the lower quartile (0≤4).

**Table 7:** Concentrations of biomarkers in all participants according to *total* seafood index quartiles

		*Total* seafood index, quartile (range)			
		
Biomarker	*n*	Q1 (0≤4)	Q2 (>4–6.7)	Q3 (>6.7–8)	Q4 (>8–9.5)
25OH vitamin D (nmol/L)†	30	41±14^b^	57±28a,b	94±23^a^	77±33^a^
EPA (mg/g RBC)‡	54	0.015±0.01^b^	0.017±0.01^b^	0.029±0.02^a^	0.032±0.02^a^
DPA (mg/g RBC)‡	54	0.049±0.01a,b	0.046±0.01^b^	0.056±0.01^a^	0.056±0.01^a^
DHA (mg/g RBC)‡	54	0.16±0.03b,c	0.16±0.04b,c	0.18±0.04^a^	0.182±0.03a,c
Omega-3 index§	54	6.1±1.5^b^	6.2±1.5^b^	7.4±1.6a,b	7.4±1.7^a^
RBC HUFA score‖	54	32±4b,c	35±3^b^	38±7a,b	39±7^a^

a,b,c Different letters indicate statistical significant difference, *P*<0.005 (ANOVA Fisher LSD post hoc test).

† Participants who answered in the winter: *n*=30.

‡ *n*=54.

§ The content of EPA+DHA in red blood cells membranes expressed as a percent of total fatty acid (13).

‖ Total HUFA is the sum of the omega-3 and the omega-6 HUFAs, and the red blood cells omega-3 HUFA score equals 100% – omega-6 HUFA (14).

## Discussion

The seafood index was developed to estimate seafood consumption in pregnant women. We observed a strong correlation between the reported and the estimated seafood consumption. This indicates that the seafood index, developed for this seafood FFQ is a valid method for data processing and interpretation. And thus, our method can be used to obtain information concerning both the total seafood consumption and the consumption of different types of seafood. The index might be an important step towards studying the health effects of seafood consumption in the future. Calculating the seafood indexes from the seafood FFQ is a good method to stratify the reported seafood intake of respondents. Our seafood FFQ estimates the omega-3 index and the RBC omega-3 HUFA score during the whole year and vitamin D intake in the winter months in a population of pregnant women in the southern part of Norway. Women in this study were primarily Caucasian Norwegians of middle and upper income, with a longer period spent in education than Norwegian women in general (32). Application of results to other pregnant groups remains to be elucidated.

In their comprehensive FFQ, Hjartaker et al. found that summary questions on lean and oily fish for dinner estimated average consumption frequency similar to the detailed item questions (33). However, their two summary questions were not able to relate fish intake to the FA composition in serum phospholipids (33). The results obtained in this study are in accordance to Hjartaker et al. regarding the summary question concerning seafood as dinner. However, in the present study we also observed a positive correlation between the summary question concerning seafood as spread which also correlation with the FA composition in RBC (all the omega-3 biomarkers analysed in RBCs). Based on this study, the *total* seafood index is a crude measure of the participants’ seafood and marine omega-3 supplement intake. Still, a good correlation was found between the *total* seafood index, all the RBC omega-3 biomarkers, and the serum 25OH vitamin D concentration. These correlations underlines the importance of including questions on the use of dietary supplements, when studying diet, as supplements are part of the *total* seafood index.

One portion (150 g) of Atlantic-farmed salmon, the main oily fish consumed for dinner (data not shown), contains 8–9 µg of vitamin D and 3 g of EPA and DHA (www.nifes.no/sjomatdata) (34). This is almost sufficient enough to provide the recommended dietary daily intake of vitamin D in pregnancy (10 µg) and provides considerably more than the recommended daily intake of EPA and DHA (250 mg/day) in healthy people set by European Food Safety Authorities (EFSA) (4, 35). There was a positive correlation between the index for oily fish for dinner and the serum 25OH vitamin D concentration. In contrast, oily fish for dinner did not correlate with the DHA concentration in the RBC, the omega-3 HUFA score, nor the omega-3 index. It is worth noticing that the results from the association between the serum 25OH vitamin D concentration and oily fish were from those participants who answered during the winter months only. Associations between the omega-3 biomarkers and oily fish are based on the entire study population. There may be confounding factors in the overlapping small groups and, as such, results need to be explored in a larger population. Circulating 25OH vitamin D probably crosses the hemochorial human placenta readily, and cord blood 25OH vitamin D concentrations are equal to or up to 20% lower than maternal concentrations (36, 37). Maternal metabolism may contribute to the lack of correlation of oily fish consumption with the DHA concentration in RBC, the omega-3 HUFA score, and the omega-3 index. To fulfil the demands of the growing foetus, pregnancy is associated with a reduction in the relative amounts of PUFA stores, especially DHA, in the maternal circulation (38). If the mother's PUFA- and DHA stores are depleted, prior to or during pregnancy, the transfer of these nutrients to the foetus will reduce the correlation between maternal seafood consumption of oily fish and maternal blood levels of PUFAs.

A positive correlation was observed between the *total* seafood spread index and the marine omega-3 biomarkers. As expected, the association between the latter and the oily fish spread index was even stronger. Our findings of an association between maternal seafood and omega-3 supplement intake, and the biomarkers of omega-3 in blood, are in close agreement with other studies (10, 20, 39, 40). However, there are only few published validation studies in pregnant women, and the studies are difficult to compare due to differences in the FFQ instruments, reference methods, various time points of assessment in pregnancy, and differences in population size.

In our study, a stronger correlation was observed between the *total* seafood index and the RBC omega-3 HUFA score, than between the *total* seafood index and the omega-3 index. The omega-3 HUFA score has previously been reported to be a better biomarker than the omega-3 index as DPA is included (27). The ability to predict the omega-3 HUFA levels in various tissues and blood fractions tends to be stronger when expressed as omega-3 HUFA score rather than the omega-3 index (27).

Pregnant women in Norway are advised to consume 5 ml cod liver oil daily (4). The supplement index (vitamin D containing omega-3 supplementation) strongly correlated with the serum 25OH vitamin D concentration in participants who answered during the winter. The *total* seafood index is a summation of the seafood dinner index, the seafood spread index, and the supplement index (Fig. 1.) Omega-3 supplementation is likely to be a strong contributor to the correlation between the crude *total* seafood index and the serum 25OH vitamin D concentration. The results elucidate the importance for pregnant women to include vitamin D supplements in the diet when exposure to sunlight is minimal at Norwegian latitudes.

In this study, the *total* seafood index ranged from 0 to 11. Biomarker concentrations increased across increasing quartiles of seafood and omega-3 supplements from the FFQ (Table 7). Participants with a *total* seafood index between 8 and 11 had a significantly higher biomarker status, including all the biomarkers analysed, than participants with a *total* seafood index between 0 and 4. In this study, the authors therefore consider a *total* seafood index of 8 or above as high, and a *total* seafood intake of 4 or lower as low.

With a small sample size, the results should be interpreted with caution, as the findings might not be valid for all populations. However, the study population was nationally representative in terms of age (41) and seafood consumption (42) but tended to omit those with no education. A consequence of using biomarkers and not a dietary assessment tool as the reference instrument, in the validation of the seafood FFQ, was that energy adjustment was not possible. However, biomarkers reflect actual rather than reported intakes and, as such, are unaffected by respondents’ reporting bias (43).

## Conclusion

We established a seafood index from the seafood FFQ, which can be used when studying health effects of seafood consumption in large populations. Based on correlation analyses, the seafood FFQ captures information on seafood consumption considerably well, in a group of pregnant women. This seafood FFQ correlates fairly well with the omega-3 index, the RBC omega-3 HUFA score, and serum 25OH vitamin D, and it distinguishes between low and high seafood consumers, regarding the above-mentioned biomarkers.
